# Plasma proteome changes linked to late phase response after inhaled allergen challenge in asthmatics

**DOI:** 10.1186/s12931-022-01968-0

**Published:** 2022-03-05

**Authors:** Maria Weitoft, Måns Kadefors, Henning Stenberg, Ellen Tufvesson, Zuzana Diamant, Sara Rolandsson Enes, Leif Bjermer, Oskar Rosmark, Gunilla Westergren-Thorsson

**Affiliations:** 1 Division of Lung Biology, Department of Experimental Medical Science, Lund University, Lund, Sweden; 2 Division of Respiratory Medicine and Allergology, Department of Clinical Sciences Lund, Lund University, Lund, Sweden; 3 Center for Primary Health Care Research, Department of Clinical Sciences Malmö, Lund University, Malmö, Sweden; 4 Department of Microbiology Immunology and Transplantation, KU Leuven, Catholic University of Leuven, Leuven, Belgium; 5 Department of Clin Pharm and Pharmacol, University of Groningen, Univ Med Ctr Groningen, Groningen, Netherlands

**Keywords:** Airway inflammation, Allergic asthma, Coagulation, Mass spectrometry, Protease inhibition

## Abstract

**Background:**

A subset of individuals with allergic asthma develops a late phase response (LPR) to inhaled allergens, which is characterized by a prolonged airway obstruction, airway inflammation and airway hyperresponsiveness. The aim of this study was to identify changes in the plasma proteome and circulating hematopoietic progenitor cells associated with the LPR following inhaled allergen challenge.

**Methods:**

Serial plasma samples from asthmatics undergoing inhaled allergen challenge were analyzed by mass spectrometry and immunosorbent assays. Peripheral blood mononuclear cells were analyzed by flow cytometry. Mass spectrometry data were analyzed using a linear regression to model the relationship between airway obstruction during the LPR and plasma proteome changes. Data from immunosorbent assays were analyzed using linear mixed models.

**Results:**

Out of 396 proteins quantified in plasma, 150 showed a statistically significant change 23 h post allergen challenge. Among the most upregulated proteins were three protease inhibitors: alpha-1-antitrypsin, alpha-1-antichymotrypsin and plasma serine protease inhibitor. Altered levels of 13 proteins were associated with the LPR, including increased factor XIII A and decreased von Willebrand factor. No relationship was found between the LPR and changes in the proportions of classical, intermediate, and non-classical monocytes.

**Conclusions:**

Allergic reactions to inhaled allergens in asthmatic subjects were associated with changes in a large proportion of the measured plasma proteome, whereof protease inhibitors showed the largest changes, likely to influence the inflammatory response. Many of the proteins altered in relation to the LPR are associated with coagulation, highlighting potential mechanistic targets for future treatments of type-2 asthma.

**Supplementary Information:**

The online version contains supplementary material available at 10.1186/s12931-022-01968-0.

## Background

Asthma is a heterogeneous disease with a diverse immunopathology reflected in various clinical phenotypes or endotypes and affects approximately 300 million people worldwide [1, 2]. There is a close association between allergy and asthma, and allergen-driven asthma is considered the most common phenotype of asthma in children and younger adults [3]. Allergen challenge of subjects with allergic asthma serves as a model to study several features of acute and chronic responses to inhaled allergens. Based on the drop in forced expiratory volume in one second (FEV_1_), allergen-induced airway responses are generally defined as an early phase response (EPR), which can be followed by a late phase response (LPR), usually starting between 4 and 8 h post allergen challenge [4]. While the EPR is an IgE-mediated, mast cell-triggered phenomenon occurring within minutes of allergen exposure and lasting up to approximately 2–3 h, the LPR is associated with the recruitment of several inflammatory leukocytes and the release of their inflammatory products. It can last for several hours to days and is associated with more chronic sequelae including airway hyperresponsiveness [5]. Approximately 50% of subjects with allergic asthma develop a LPR, which affects small airways to a larger extent, indicative of a more extensive airway pathology [6]. Airway remodeling, such as subepithelial fibrosis, correlates with the number of circulating fibrocytes in asthma [7, 8]. Circulating fibrocytes have been described to derive from CD34^+^ hematopoietic pool of progenitors or/and from a subset of CD14^+^ CD16^−^ group of monocytes [9, 10]. This possible source of structural cells is recruited from the circulation into the tissue through the SDF-1/CXCR4 axis upon interaction with hyaluronan and associates with platelet activation and blood clotting [11–13].

The molecular mechanisms underlying the LPR, including the recruitment of hematopoietic progenitors, are still not fully clarified. In this study, we studied plasma samples and peripheral blood mononuclear cells (PBMCs) from subjects with an allergen induced EPR with varying degrees of LPR following an inhaled allergen challenge [6]. The aim was to investigate changes in proteome profile and hematopoietic progenitor cell recruitment related to the LPR, represented by the maximal decrease in FEV_1_ 4–8 h post allergen challenge (late FEV_1_ drop). Consequently, we observed distinguishing factors associated with a LPR suggestive of new mechanisms, which warrant further research that can contribute to the development of targeted treatment strategies.

## Materials and methods

### Subjects and study design

**Fig. 1:**
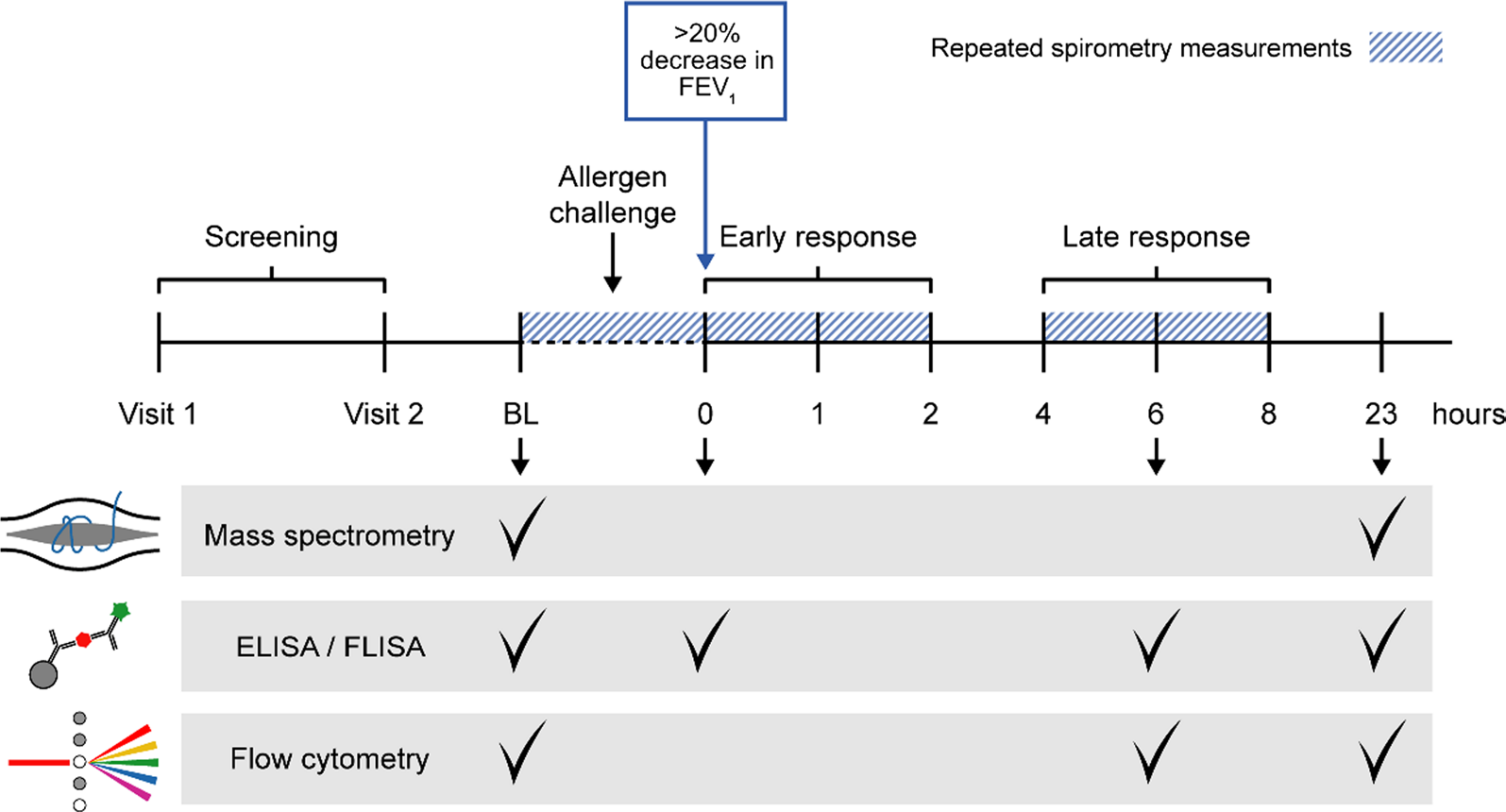
Study overview. At visit 1, subjects’ eligibility to participate in the study was assessed by a skin prick test consisting of 10 common aeroallergens, specific IgE levels in serum and a methacholine challenge test. Baseline lung physiology assessments were conducted before and at predefined timepoints following an inhaled allergen challenge. The allergen challenge was considered complete when a > 20% decrease in FEV_1_ was achieved. The timeline indicates when blood/plasma samples were collected for the respective analyses in relation to the completion of the allergen challenge

An overview of the study design is shown in Fig. 1. The material was collected from a larger trial focusing on lung physiology and biomarkers [6]. In total, 32 subjects with allergic asthma were included in the study (subject characteristics listed in Table 1, details in Additional file 1: Table S1 including subject allocation to the different analyses). All subjects were in overall good health with no respiratory infection within 3 weeks before screening, confirmed a clinically stable asthma. None of the subjects were smokers (within 1 year before screening or > 10 pack-years) or had received treatment with leukotriene or muscarinic receptor antagonists, phosphodiesterase inhibitors, oral corticosteroids, biologicals (e.g., anti-IgE, anti-IL5) or allergen-specific immunotherapy within 6 months before screening. Approximately 50% of subjects were on a stable dose of ICS (≤ 400 μg budesonide/day) for at least 3 months prior to the study, which remained unchanged throughout the study (Table 1). Short- and long-acting beta2-agonists were withheld for 8 h and 48 h, respectively, prior to any study-related procedures.

**Table 1:** Subject characteristics

	All (n = 32)	Single responders (n = 18)	Dual responders (n = 14)
Sex (F/M)	16/16	9/9	7/7
Age (y)	27 (24–37)	27 (26–42)*	24 (22–30)*
BMI	24 (22–26)	23 (22–26)	24 (23–26)
Regular ICS use (n)	15	6	9
FEV1 Baseline (% predicted)	96 (90–104)	95 (88–103)	95 (92–105)
Early drop in FEV1 (%)	23 (21–25)	22 (21–25)	23 (21–25)
Max. drop in FEV1 after 4–8 h (%)	10 (7–17)	7 (5–9)	20 (15–24)
Allergen used in challenge (n) (Cat/Horse/HDM/ Birch/Grass)	17/6/2/4/3	10/4/1/2/1	7/2/1/2/2

Data presented as median (IQR). BMI = body mass index, FEV1 = forced expiratory volume in 1 s, ICS = inhaled glucocorticosteroid (maximal dose of 400 μg Budesonide/day)

Statistical differences between single and dual responders were analysed using non-parametric Mann–Whitney, *denotes p < 0.05

Subjects were screened on two separate screening visits (Fig. 1) with methacholine and mannitol challenge performed as previously described [6]. Eligible subjects underwent a standardized titrated inhaled allergen challenge with a sensitizing allergen (based on subject history and allergen testing), as previously described [6]. Flow volume spirometry was conducted with a Jaeger MasterScope (Erich Jaeger GmbH), at baseline (BL) and predefined time points following allergen challenge, in accordance with European Respiratory Society (ERS)/American Thoracic Society (ATS) standards [14].

For allocation of subjects for flow cytometry and immunosorbent assays, subjects were designated as either single responders (SR) or dual responders (DR), where the latter had a decrease in FEV_1_ > 12% 4–8 h post allergen challenge (Fig. 1). SR and DR differed in age and ICS use, but not in any other variable, including BL FEV_1_, duration of asthma, ACT-score, methacholine PD20, mannitol PD15, total IgE levels, serum specific IgE levels, SPT wheal diameter or number of sensitizations. Additional subject characteristics have previously been published [6]. Blood samples for analysis of plasma components (proteins and glycosaminoglycans) were collected in EDTA tubes before allergen challenge (BL), 0 and 0.5, 1, 2, 6, 8 and 23 h after the allergen challenge [15]. Blood samples for preparation of PBMCs were collected in heparin tubes at BL, as well as 6 and 23 h after allergen challenge.

### Liquid chromatography–mass spectrometry analysis of plasma samples

Experimental details are provided in the supporting information. In brief, reduced and alkylated plasma protein samples were digested with Lys C followed by trypsin, desalted and spiked with retention time peptides before injection of 1 µg peptide/sample. Analysis was performed on a QExactive HF-X (Thermo Fischer Scientific) mass spectrometer operated in data-independent acquisition (DIA) mode. DIA data were analyzed by using Spectronaut against a plasma spectral library provided by Wåhlén et al. (manuscript in preparation). An FDR of 0.01 using Q value were employed for both precursor and protein identification. The data were exported from Spectronaut and downstream statistical analysis was performed in R (version 4.0.2). The mass spectrometry (MS) proteomics data have been deposited to the ProteomeXchange Consortium via the PRIDE [16] partner repository with the dataset identifier PXD027091.

### DIA data normalization and processing

Proteins quantified in less than half of the samples at each timepoint and common contaminants were excluded from further analysis. Protein quantity values were normalized by dividing the value for each protein with the summed intensities for all quantified proteins in the respective sample, followed by log^2^ transformation. Normalized data were analyzed using the DEqMS software package in R including timepoint (BL or 23 h), sex, BMI and age as factors for a linear model [17]. Statistics for the differences between BL and 23 h were extracted. A separate model was fitted where the difference between BL and 23 h protein was analyzed against the same factors, but with late FEV_1_ drop in place of timepoint. Inclusion of ICS dose in the models had limited impact on the results and was left out to avoid overfitting.

### Functional enrichment analysis

We used STRING [18] and Gene Ontology [19, 20] to perform a functional enrichment analysis for the significantly altered proteins after allergen challenge. Over-representation analysis of protein groups related to biological process terms was performed with the FDR set to < 5%, using the Benjamani-Hochberg method.

### Luminex assay for quantification of selected plasma proteins

Five of the significantly altered proteins in the MS analysis were manually selected for luminex analysis, based on change between BL and 23 h for the entire study population, or late FEV_1_ drop. The selection was made based on conceived pathophysiological interest. Plasma samples were from timepoints BL, 0 min (EPR), 6 h (LPR), and 23 h, from 10 SR and 10 DR. Proteins were quantified according to the manufacturers’ instructions on a Luminex MAGPIX instrument (Luminex Human Magnetic Assay, R&D Systems).

### Hyaluronan and SDF-1 ELISA

Total plasma hyaluronan (n = 18) and SDF-1 (n = 6) were quantified using two commercially available ELISA kits (R&D Systems, Hyaluronan Quantikine ELISA Kit, DHYAL0, Human CXCL12/SDF-1 alpha Quantikine ELISA Kit, DSA00).

### Statistical analysis of FLISA and ELISA data

We used R (version 4.0.2) and lme4 [21] to perform a linear mixed effects analysis of how protein quantification values from FLISA and ELISA measurements varied depending on time after allergen challenge and late FEV_1_ drop. One model was fitted for each protein and values from FLISA measurements were log^2^ transformed before analysis. Age, BMI, use of ICS and sex were entered into the model as fixed effects, without interaction terms. Random effects consisted of random intercepts for subjects. No obvious deviation from normality or homoscedasticity were found by visual inspection of residual plots. p-values and confidence intervals from the lme4 models were obtained using the R package sjPlot [22] employing the Kenward-Roger approximation for the degrees of freedom. Plots were generated using GraphPad Prism version 8.3.0.

### Flow cytometric analysis

Experimental details on PBMC isolation, flow cytometry staining and analysis are provided in the supporting information.

## Results

### Plasma proteome changes persist 23 h after inhaled allergen challenge

**Fig. 2:**
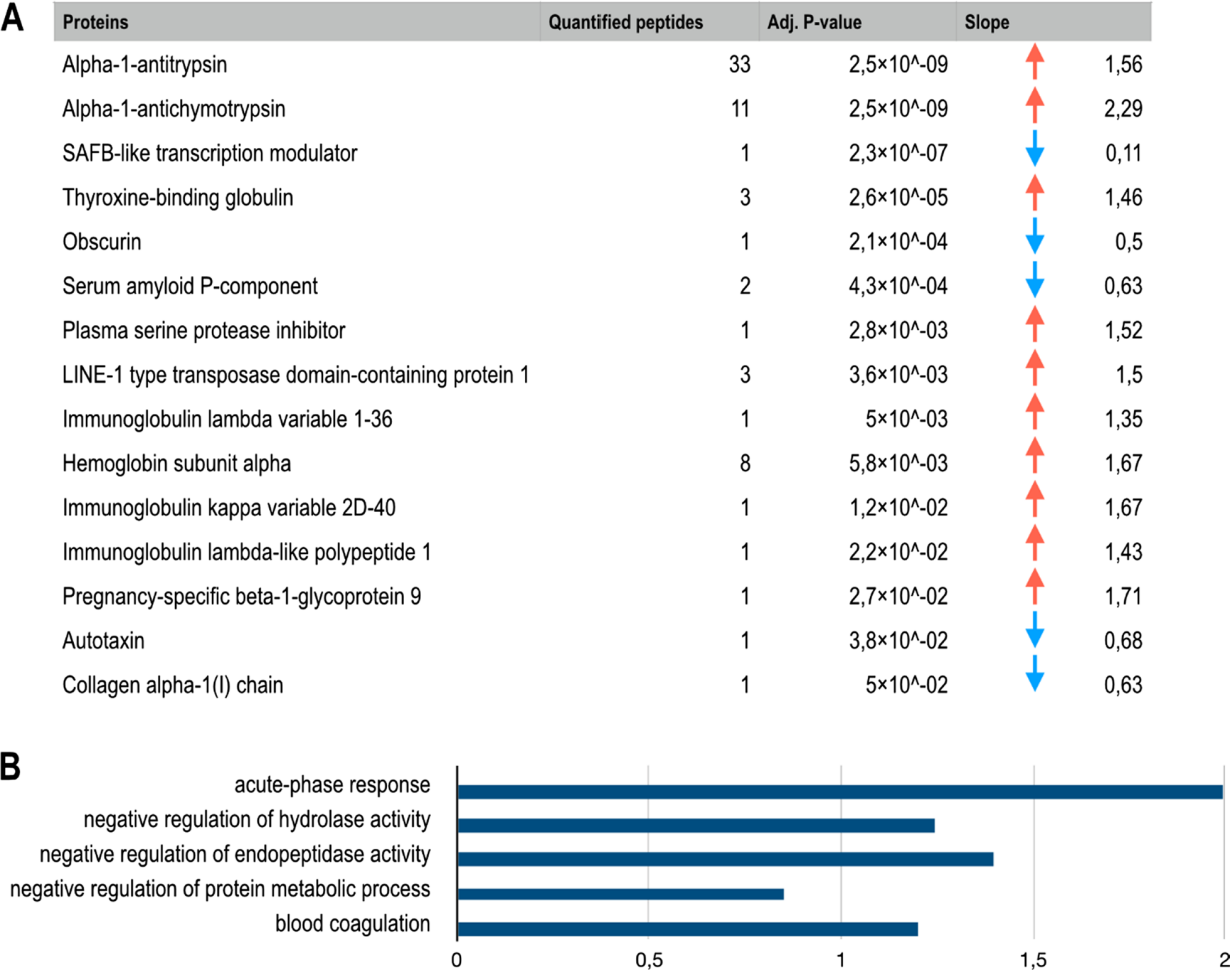
Plasma proteins altered 23 h post allergen challenge in subjects with asthma. **A** Proteins with a statistically significant change from BL to 23 h post allergen challenge that are altered > 30% compared to BL levels, according to DEqMS analysis. **B** Gene ontology terms that are significantly overrepresented (p < 0.05) for the proteins in **A,** terms are sorted by decreasing p-value, x-axis show the size of the enrichment effect [Log10(observed/expected)]

To identify plasma proteome alterations associated with allergic reaction, samples from BL and 23 h post allergen provocation were analyzed using label-free DIA-MS. A total of 396 plasma proteins were quantified, whereof 84 were immunoglobulin chains. When comparing plasma proteome profiles based on allergen provocation from BL and 23 h we identified 150 proteins that statistically significantly differed (adjusted p-value < 0.05), of which 15 proteins had a > 30% change from BL to 23 h (Fig. 2A, Additional file 1: Fig. S1). Among these 15 proteins, three protease inhibitors showed consistent and substantial increases 23 h post-allergen challenge, i.e. alpha-1-antitrypsin, alpha-1-antichymotrypsin and plasma serine protease inhibitor (Fig. 2A). In contrast, SAFB-like transcription modulator, autotaxin, obscurin, serum amyloid P (SAP)-component and collagen alpha-1(I) chain were among the proteins showing significant decreases 23 h post-allergen challenge (Fig. 2A).

### Specific plasma proteome changes are associated with the LPR

**Fig. 3:**
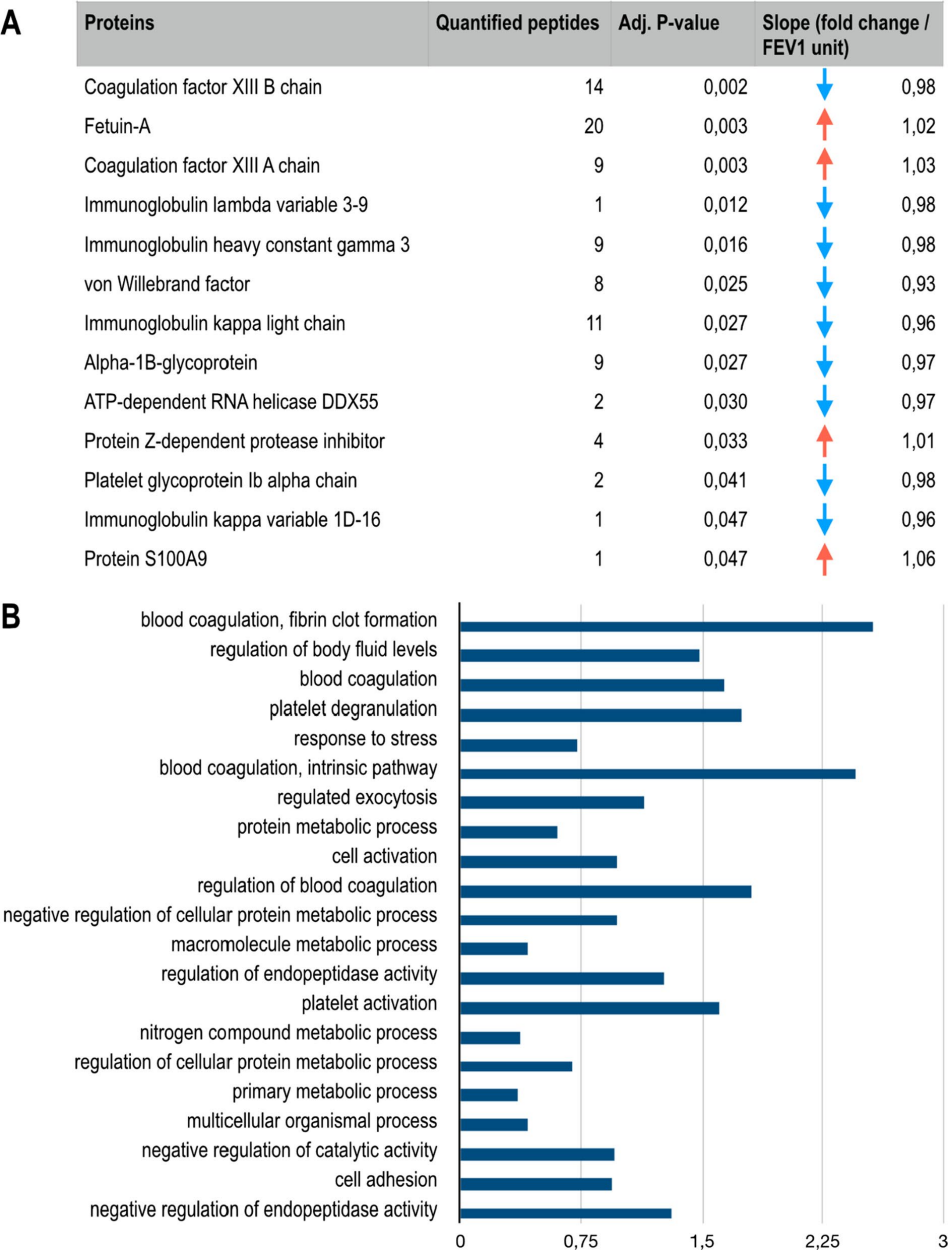
Plasma proteins with a change from BL to 23 h post allergen challenge that are associated with the LPR, approximated by the maximal drop in FEV_1_ 4–8 h post allergen challenge. **A** Table of proteins with a statistically significant change from BL to 23 h post allergen challenge that correlate with the drop in FEV_1_ during the LPR, according to DEqMS analysis. **B** Gene ontology terms that are significantly overrepresented (p < 0.05) for the proteins in **A**, terms are sorted by increasing p-value, x-axis shows the size of the enrichment effect [Log10(observed/expected)]

To find proteome alteration associated with the LPR, change in protein levels between BL and 23 h (delta values) were analyzed against late FEV_1_ drop (adjusted for sex, BMI and age), which identified 13 proteins that were significantly altered based on late FEV_1_ drop, and thus degree of LPR (Fig. 3A). The change in levels for Fetuin-A, coagulation factor XIII A chain, protein Z-dependent protease inhibitor and protein S100A9 all had a positive correlation with the size of the LPR. Among the proteins for which decreasing levels correlated with late FEV_1_ drop were four proteins linked to platelet activation and blood clot formation: von Willebrand factor, alpha-1B-glycoprotein, platelet glycoprotein Ib alpha chain and coagulation factor XIII B chain (Fig. 3A, Additional file 1: Fig. S2).

### Functional enrichment analysis of significantly altered proteins according to MS-analysis

To explore protein–protein interactions and to identify potentially enriched pathways following allergen challenge, we used String database and Gene Ontology to examine protein–protein interactions and to perform an enrichment analysis of proteins with significantly altered abundance after allergen provocation. There were five pathways associated with the altered protein profile after allergen challenge irrespective of late FEV_1_ drop. Significantly enriched pathways included the acute phase response, negative regulation of hydrolase activity, negative regulation of endopeptidase activity, negative regulation of protein metabolic process, and blood coagulation (Fig. 2B). Based on late FEV_1_ drop, there were 21 significantly enriched pathways, including several pathways involved in regulation of blood coagulation and fibrin clot formation as well as regulation of metabolic processes (Fig. 3B).

### Allergen induced changes in levels of pro- and anti-inflammatory proteins normalize within 24 h

**Fig. 4:**
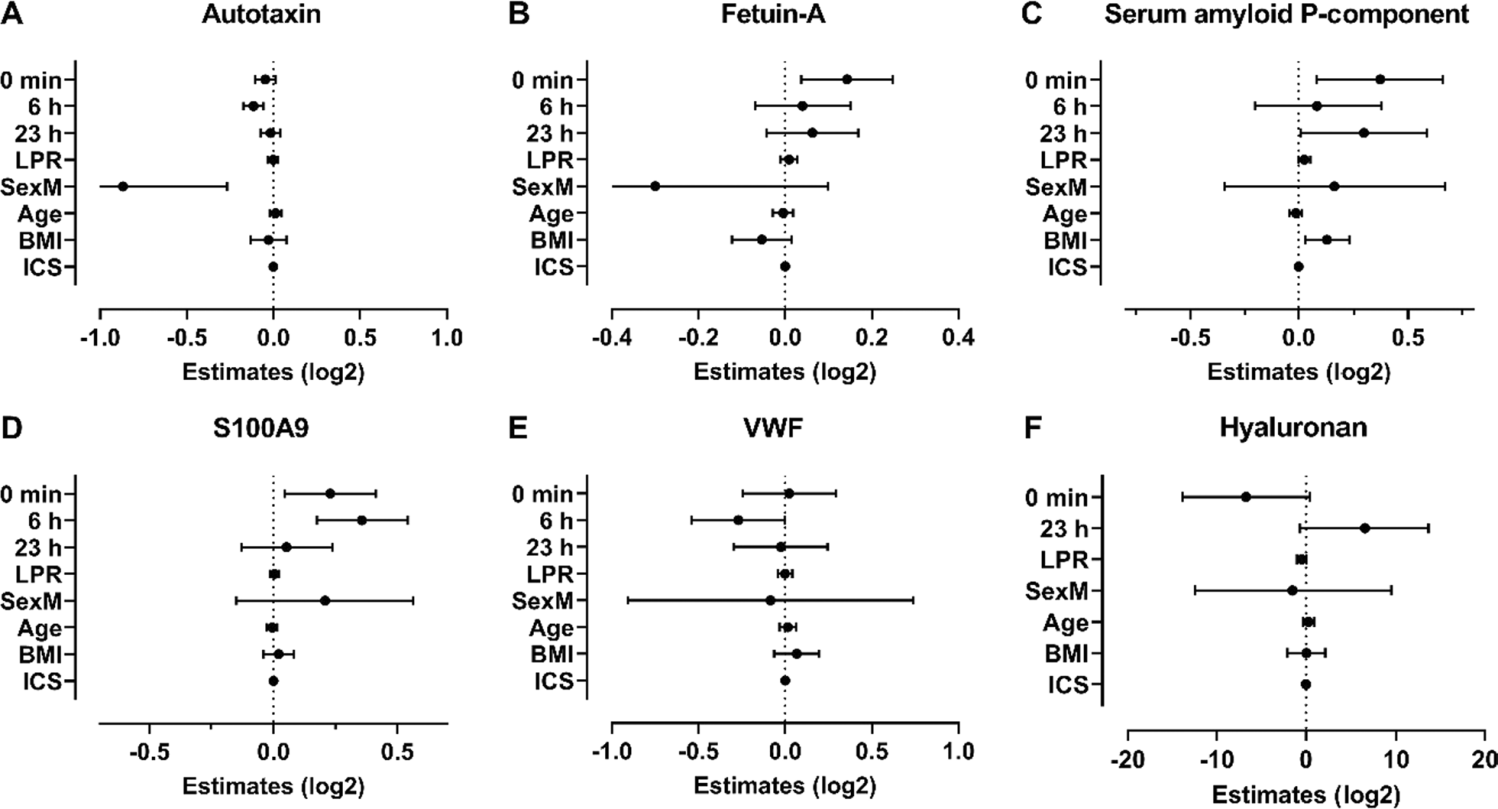
Plasma composition changes following inhaled allergen challenge. Proteins in plasma samples were analyzed using a multiplex fluorophore linked immunosorbent assay (FLISA/Luminex) and hyaluronan were analyzed by ELISA. Statistical analysis was performed using linear mixed models. The time points 0 min, 6 h and 23 h are compared to BL values, LPR represents change per % of late FEV_1_, SexM represents the effect of male sex where the point estimate for females constitutes the reference

To further study the proteins identified in the MS analysis SAP, autotaxin, fetuin-A, VWF, and S100A9 in plasma samples were analyzed with Luminex from 10 SRs and 10 DRs, at time points BL, 0 (EPR), 6 (LPR) and 23 h. Autotaxin was decreased by 8% compared to BL at 6 h post allergen challenge (95% CI − 4 to − 11%), with a tendency towards a decrease also at 0 min and 23 h post-allergen challenge (Fig. 4A). Autotaxin was influenced by sex (45% lower levels in males, 95% CI − 17 to − 64%), in accordance with published data [23]. Fetuin-A was increased by 10% at 0 min (95% CI 3–19%) followed by smaller non-significant increases at 6 and 23 h post allergen challenge (Fig. 4B). SAP was increased by 29% at 0 min (95% CI 6–58%) followed by a non-significant increase compared to BL both at 6 and 23 h post-allergen challenge (Fig. 4C). S100A9 was increased by 17% at 0 min (95% CI 3–33%) and 28% at 6 h post allergen challenge (95% CI 12–45%, Fig. 4D). VWF had a tendency towards a decrease 6 h post provocation (95% CI − 0.02 to − 31%, Fig. 4E). Collectively, four of the five analyzed proteins exhibited a change at least at one of the examined time points following allergen challenge. These four proteins have been suggested to be involved in the inflammatory reaction in asthma, fetuin considered mainly anti-inflammatory and the other three mainly pro-inflammatory [24–27]. None of the examined proteins had a significant association with the magnitude of the LPR or ICS use in this analysis.

### Temporal profiles of hyaluronan and SDF-1 analyzed by ELISA

Hyaluronan and SDF-1 are both known to play a role in the recruitment of inflammatory cells and therefore levels of hyaluronan and SDF-1 in plasma samples from SR and DR subjects were measured using ELISA. Plasma hyaluronan levels showed a non-significant decrease at 0 min compared to BL (95% CI − 47 to 2%), with a subsequent non-significant increase 23 h post allergen challenge (95% CI − 2 to 47%), which could indicate a rapid turnover of the carbohydrate chain (Fig. 4F). There was no obvious association between hyaluronan levels in plasma and the magnitude of the LPR. The SDF-1 analysis yielded results with large variance where no obvious pattern neither in relation to the temporal profile nor degree of LPR as assessed comparing SR and DR (Additional file 1: Fig. S3).

### No significant differences in proportions of classical, intermediate, and non-classical monocytes between single or dual responders

**Fig. 5:**
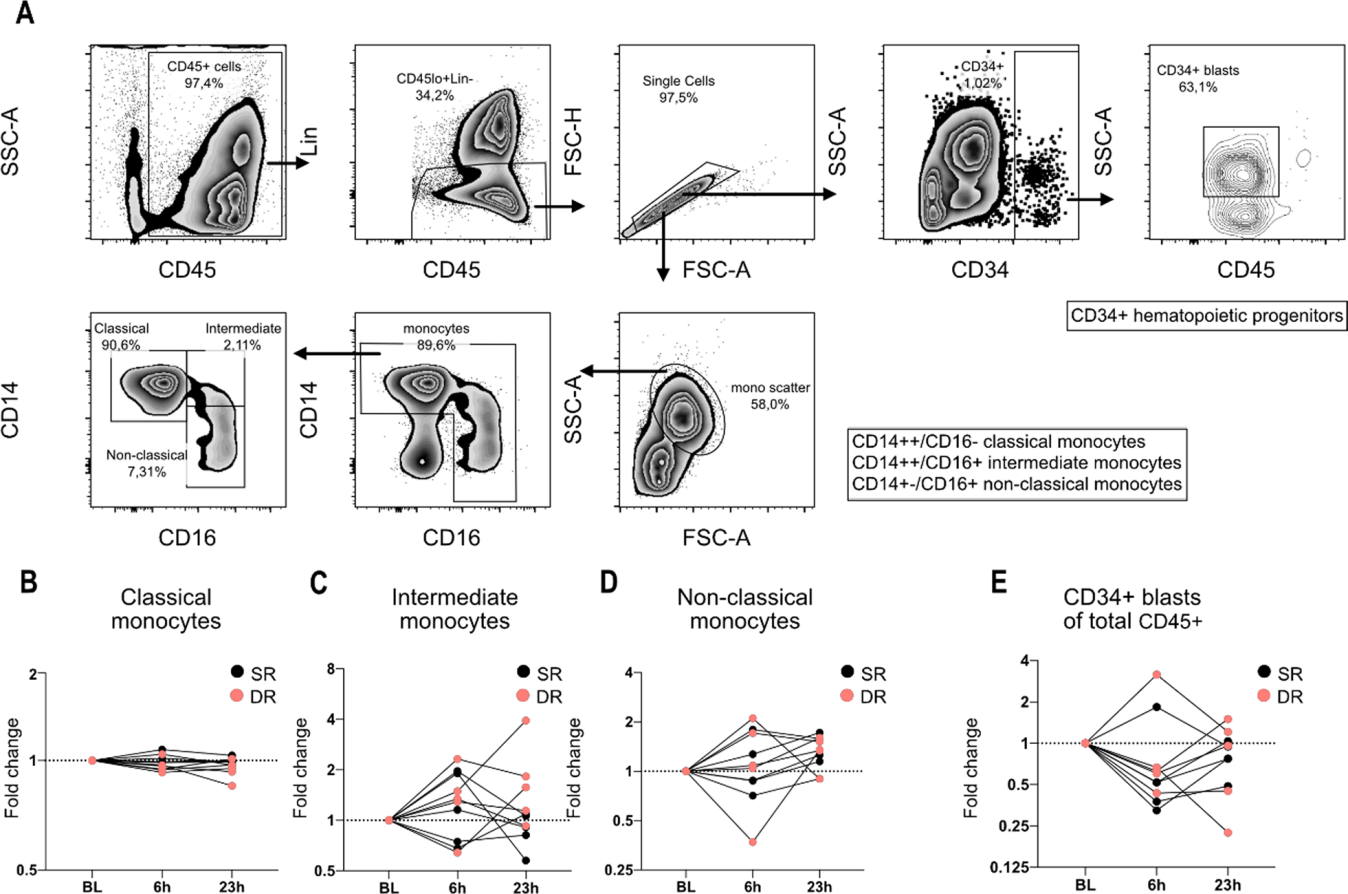
Flow cytometry analysis of circulating monocytes and hematopoietic progenitors in blood from 5 SRs and 5 DRs. **A** Gating strategy used for identification of CD34 + hematopoietic progenitors (CD34 + blasts) and monocyte subsets (classical CD14^bright^/CD16^−^, intermediate CD14^bright^/CD16^+^ and non-classical CD14^−/dim^/CD16^+^). **B**–**D** Changes in frequencies of circulating monocyte subsets out of all monocytes over time for classical (**A**), intermediate (**B**) and non-classical (**C**) monocytes. **D** Changes in frequencies of circulating CD34 + progenitors out of all CD45 + cells. Cell frequency data are presented as fold change from BL for each subject. Black dots represent single responders (SR) and red dots represent dual responders (DR)

To assess the effects of allergen challenge on the recruitment of circulating hematopoietic progenitors, blood samples were analyzed by flow cytometry. First, we identified circulating monocyte subsets and CD34 + hematopoietic progenitors at BL, and 6 and 23 h after allergen challenge from five SR and five DR. Representative data showing the flow cytometry gating strategy for monocyte subsets and CD34 + hematopoietic progenitors are shown in (Fig. 5A).

Within the monocyte population (CD45^+^/CD3^−^/CD294^−^/CD20^−^/CD19^−^/CD14^+^/CD16^+^) we quantified classical (CD14^bright^/CD16^−^), intermediate (CD14^bright^/CD16^+^) and non-classical (CD14^−/dim^/CD16^+^) monocyte subsets. There was no significant difference in monocyte subset numbers when comparing time points or SR versus DR (Fig. 5B–D). The proportions of the subtypes; classical (89.7%), intermediate (3.30%) and non-classical (6.84%) monocytes were similar to numbers reported by Sampath et al. [28]. We also examined the expression of CXC chemokine receptor 4 (CXCR4) on monocyte subsets, a receptor that is involved in the recruitment of hematopoietic progenitors [29, 30]. CXCR4 expression was 7.14 times higher in classical monocytes (median ΔMFI 1400) compared to non-classical monocytes (median ΔMFI 196). We could not detect any difference in CXCR4 expression between time points or between SR and DR (data not shown). Quantification of circulating CD34 + hematopoietic progenitors (CD34^bright^/CD45^dim^) showed a non-significant decrease in frequency during the LPR (Fig. 5A and E).

## Discussion

In this study plasma proteomics were applied to investigate pathways underlying the pathology of the LPR. In previous studies, including from our group, LPR has been shown to be associated with prolonged increases in clinical signs and symptoms, airway inflammation, airway hyperresponsiveness, small airways dysfunction and structural changes [4, 6, 31–34]. Proteomics data were analyzed by fitting a linear model, which identified 13 proteins linked to the development of a LPR, in addition, 150 proteins were significantly altered in abundance 23 h after allergen challenge irrespective of the LPR.

Pathway analysis of the proteins most affected by allergen challenge i.e., > 30% change from BL values, showed enrichment for acute phase response, blood coagulation and protease inhibition. Three protease inhibitors were among those showing the largest change, i.e., alpha-1-antitrypsin, alpha-1-antichymotrypsin and plasma serine protease inhibitor, all showing a > 50% increase from BL, possibly reflecting an active regulation of inflammation induced protease activity. Alpha-1-antitrypsin, alpha-1-antichymotrypsin and the downregulated protein SAP are all significantly altered acute phase proteins [35]. SAP belongs to the pentraxin family of proteins together with the widely used clinical marker of inflammation, C-reactive protein (CRP); the latter not showing a significant change in our MS-data. SAP has been shown to inhibit neutrophil adhesion to extracellular matrix, differentiation of hematopoietic myeloid progenitors into fibrocytes and to promote the formation of immune-regulatory macrophages [36, 37]. In the context of a response to inhaled allergen, SAP may have a protective role by driving monocyte differentiation towards the anti-inflammatory and anti-fibrotic M2c phenotype [38, 39].

Autotaxin is a secreted enzyme that produces most of the extracellular lipid mediator lysophosphatidic acid (LPA), that promote chemotaxis of Th2 cells and IL-13 expression [40]. A previous study showed increased sputum levels of autotaxin in allergic asthmatics following a segmental allergen challenge [24]. We found decreased plasma levels of autotaxin, a discrepancy possibly explained by a negative feedback mechanism, where high autotaxin activity in the lung could inhibit systemic expression of autotaxin measured in plasma via LPA, or the related lipid product sphingosine 1-phosphate [41].

The LPR associated proteins showed enrichment for biological processes related to hemostasis, which agrees with existing literature linking coagulation factors in sputum and plasma from subjects with asthma with an ongoing inflammatory process [42, 43]. Two of these proteins, glycoprotein Ib alpha chain and VWF, work in concert to promote platelet adhesion and activation, the latter has also been found to be an important pro-inflammatory mediator involved in leukocyte extravasation [44]. Both proteins showed an inverse correlation to the late FEV_1_ drop in. Plasma VWF levels have been proposed to correlate to systemic inflammation [45].

Coagulation factor XIII is a transglutaminase known to stabilize fibrin clots as the final enzyme in the coagulation cascade [46]. Our data showed a positive correlation between the degree of LPR and levels of the catalytically active A subunit of coagulation factor XIII, and a negative correlation between LPR and the B subunit that serves a carrier/inhibitory role in the circulating pro-transglutaminase. A previous study has shown increased expression of coagulation factor XIII A in bronchoalveolar lavage after segmental allergen challenge and in induced sputum from patients with allergic asthma, with an association to type 2 inflammation and airway obstruction [48]. The functional role of coagulation factor XIII A is complex, as it has both intracellular- and extracellular functions beyond its role in coagulation e.g., serving to cross-link extracellular matrix proteins [46].

Our finding of a positive correlation between LPR and levels of protein S100A9 fits well with increased inflammation. S100A9 is constitutively expressed in immune cells, such as monocytes and neutrophils, and is released in response to environmental triggers and cellular damage [27]. A similar positive correlation was also found for fetuin-A, which has a dual role as a both positive and negative acute phase protein, depending on the mode of inflammation. High serum levels of sialylated fetuin-A have been shown to be predictive of clinical responsiveness to grass pollen immunotherapy [25, 49, 50].

Hyaluronan fragments have previously been shown to enhance SDF-1 mediated migration of bone marrow derived CD34 + progenitor cells [11]. In our study, we were not able to detect any specific pattern regarding SDF-1 levels post allergen challenge, given the large inter-subject variation and the limited number of subjects in the analysis. Hyaluronan showed a statistically non-significant decrease during the EPR. Despite no apparent effect on SDF-1, we saw a non-significant trend towards a decrease in circulating CD34 + progenitors during the LPR, which connects to a study by Schmidt et al., where an increase in fibrocytes was shown in the bronchial submucosa already 4 h after allergen exposure [51].

Finally, an obvious limitation to this study is the limited number of subjects, which limit statistical power. However, to lower the impact of inter-subject variability, subjects served as their own controls (BL values) and multilevel models was used to simultaneously analyze all proteins, which should result in an efficient effect estimation and better control of the so-called type I error [52]. A higher proportion of DR than SR used ICS, although, according to our statistical models, this was not associated with any significant effect on protein levels.

## Conclusion

This explorative study on changes in the plasma proteome associated with EPR and LPR following inhaled allergen challenge may serve as a basis for finding new druggable asthma mechanisms and open new avenues for patient stratification, facilitating targeted therapies. Protease inhibitors are upregulated post allergen challenge, potentially mitigating the effect of inflammation-related proteases. We found distinguishing plasma proteome changes in subjects experiencing a pronounced late phase response following allergen exposure. Several of these proteins are linked to coagulation, and further studies on the role of coagulation factors in allergic inflammation are warranted.

## Supplementary Information

**Additional file 1.** Supporting information.

## Data Availability

The mass spectrometry (MS) proteomics datasets generated and analysed during the current study are available in the ProteomeXchange Consortium via the PRIDE [16] partner repository with the dataset identifier PXD027091.

## Abbreviations

BL: Baseline
CD: Cluster of differentiation
CXCR4: C-X-C motif chemokine receptor 4
DR: Dual responder
ELISA: Enzyme-linked immunosorbent assay
EPR: Early phase response
FEV1: Forced expiratory volume in one second
FLISA: Fluorescence-linked immunosorbent assay
FSC: Forward scatter
MFI: Median fluorescence intensity
ICS: Inhaled corticosteroids
LPR: Late phase response
MS: Mass spectrometry
PBMC: Peripheral blood mononuclear cell
SDF-1: Stromal cell-derived factor 1
SR: Single responder
SSC: Side scatter
