# Supplementary material for: Plasma proteome changes linked to late phase response after inhaled allergen challenge in asthmatics
Source: Respir Res. 2022 Mar 5;23:50. doi: 10.1186/s12931-022-01968-0 (PMC8897854; doi:10.1186/s12931-022-01968-0)
Supplement: Supplementary file 1 — Additional file 1. Supporting information. [file 12931_2022_1968_MOESM1_ESM.pdf]

Plasma proteome changes linked to late phase response after inhaled allergen challenge in asthmatics

Supporting information

Methods

Preparation of plasma samples for mass spectrometry analysis

LC-MS proteomics

Isolation, freezing and thawing of PBMC

Flow cytometry sample processing and analysis

Flow cytometry analysis

Tables

Supplementary table 1

Supplementary table 2

Figures

Supplementary figure 1

Supplementary figure 2

Supplementary figure 3

Methods

Preparation of plasma samples for mass spectrometry analysis

Sample preparation was performed on the Agilent AssayMAP Bravo Platform. Ten microliters 1:10 diluted plasma sample was transferred to a 96-well plate (Greiner G650201) and 4 M Urea (Sigma-Aldrich) in 100 mM AmBic (Sigma-Aldrich) was added to a final volume of 50 µL. Proteins were reduced with 60 mM dithiothreitol (DTT, final concentration of 10 mM, Sigma-Aldrich) for 1 hour at 37°C followed by alkylation with 80 mM iodoacetamide (IAA, final concentration of 20 mM, Sigma-Aldrich) for 30 min in the dark at room temperature. Plasma samples were digested with Lys-C (FUJIFILM Wako Chemicals U.S.A. Corporation) at an enzyme: protein ratio of 1:50 w/w for 5 hours at room temperature and further digested with trypsin (Sequencing Grade Modified, Promega) at a trypsin: protein ratio of 1:50 w/w overnight at room temperature [16].

The digestion was stopped by adding 10% trifluoroacetic acid (TFA, Sigma-Aldrich) and the digested peptides were desalted on the Bravo platform. Ninety percent acetonitrile (ACN, Sigma-Aldrich) with 0.1% TFA and 0.1% TFA were used to prime and equilibrate the AssayMAP C18 cartridges (Agilent, PN: 5190-6532), respectively. Samples were loaded into the cartridges at a flow rate of 5 µL/min. The cartridges were washed with 0.1% TFA before peptides were eluted with 80% ACN/0.1% TFA. The eluted peptides were dried in a SpeedVac (Eppendorf) and resuspended in 25 µL of 2% ACN/0.1% TFA. The peptide concentration was measured using the Nanodrop (DeNovix Inc.). The samples were diluted to 0.5 µg/µL with 2% ACN/0.1% TFA and spiked with synthetic iRT peptides (JPT Peptide Technologies) before one µg µg of peptides was injected to the LC MS for analysis.

LC-MS proteomics

Plasma samples were analyzed on Hybrid mass spectrometer QExactive HF-X (Thermo Fischer Scientific) coupled with an EASY-nLC 1200 system (Thermo Fischer Scientific). A two-column setup was used on the HPLC system and peptides were loaded into an Acclaim PepMap 100 C18 precolumn (75 µm x 2 cm, Thermo Scientific) and subsequently separated on an EASY spray column (75 µm x 50 cm, nanoViper, C18, 2 µm, 100 Å) with the flow rate 350 nL/min. The column temperature was set to 45°C. Solvent A (0.1% FA in water) and solvent B (0.1% FA in 80% ACN) were used to create a 90 min linear gradient from 3 to 38% of solvent B in solvent A for peptide elution. The QExactive HF-X was operated in the data-independent acquisition (DIA) mode and the instrument method was adopted from previous published work [17] and optimized for 90 min gradient analysis. Full MS survey scans from m/z 350-1650 with a resolution 120,000 were performed . The automatic gain control (AGC) target was set to 3×10^6 with the maximum injection time of 60 ms. One segment for MS1 was kept constant. Forty-four segments with variable isolation windows were acquired for MS2 with the resolution of 30,000. The stepped normalized collision energy (NCE) for higher-energy collisional dissociation (HCD) was set 25.5, 27 and 30 keV and the AGC target for MS2 was 3×10^6. The maximum injection time was set to auto.

DIA data were analyzed by using Spectronaut against a plasma spectral library provided by Wåhlén et al (manuscript in preparation). The data was extracted based on the maximum intensity for both precursors and fragment ions. The default settings were applied for the peptides and proteins identification and quantification. In brief, excluding duplicate assay; generation decoy based on mutated method at 10% of library size; and estimation of FDRs using Q value as 0.01 for both precursors and proteins were performed for identification. The p-value was calculated by kernel-density estimator. For the quantification, interference correction was activated and a minimum of 3 fragment ions and 2 precursor ions were kept.

Isolation, freezing and thawing of PBMC

PBMCs were isolated using Ficoll-Paque plus according to the manufacturer's instructions (GE Healthcare). After isolation, cells were frozen in equal volumes of StemMACS MSC expansion medium (Miltenyi Biotec) and Dulbecco's phosphate-buffered saline (supplemented with 15 % DMSO, 50 % fetal bovine serum and 0.2 % Heparin (10.000 I.U.)) at -80°C and then transferred to liquid nitrogen until further analysis. Before analysis, samples were thawed for approximately 50 seconds in a 37°C water bath, then transferred to a 50 mL vial where warm medium was added dropwise during 60 seconds, with subsequent washing before further processing.

Flow cytometry sample processing and analysis

Cells were blocked with FcBlock (BD Bioscience) and then labeled with monoclonal antibodies against; CD34, CD45, CD11b, CD3, CD19, CD20, CD14, CD294, CD115, CD16, and CXCR4 (CD184) (BD Bioscience). More detailed information on all antibodies used is provided in Supplementary table 2. Since antibodies conjugated to brilliant violet conjugates were used, Brilliant Stain buffer (BD Bioscience) was added to improve staining quality. After staining of cell surface markers, cells were fixed and permeabilized with BD Cytofix/Cytoperm (BD Bioscience) before antibody staining against collagen-1 (Southern Biotech). Data from the collagen-1 staining was excluded from subsequent analysis due to uncertainty regarding the specificity of the antibody. Cells were run on a BD LSR Fortessa (BD Bioscience) and analyzed with Diva software 8.1 (BD Bioscience). Data analysis was performed using FlowJo software 10.7.1 (BD Bioscience).

Flow cytometry analysis

Cells were run on a BD LSR Fortessa (BD Bioscience) and analyzed with Diva software 8.1 (BD Bioscience). Data analysis was performed using FlowJo software 10.7.1 (BD Bioscience). Fluorescence-minus-one (FMO) stained samples were used as controls. Prior to gating of monocytes and CD34+ hematopoietic progenitors, lineage (Lin) negative lymphocytes were first identified by a CD45+ gate, followed Lin- gate on a combined dump channel (CD3, CD19, CD20 and CD294) excluding T cells, B cells, eosinophils and basophils. Doublets were excluded based on area versus height profile in forward scatter. Monocytes were identified by a high forward scatter and high side scatter, followed by a CD14/CD16 gate excluding CD14-/CD16- cells. Monocyte subsets were further identified by a CD14bright/CD16- gate (classical), a CD14bright/CD16+ gate (intermediate) and a CD14-/dim/CD16+ gate (non-classical) within the CD14/CD16 monocyte gate. CD34+ hematopoietic progenitors were identified by a CD34bright gate, followed by a CD45dim and intermediate side scatter gate.

Supplementary table 1. Subject characteristics and allocation to analyses.

Subject	Sex	Age (y)	BMI	FEV_1 Baseline (% predicted)	Budesonide (µg/day)	Early drop in FEV_1 (%)	Max. drop in FEV_1 after 4-8 h (%)	MS	Luminex	FACS	HA	SDF-1
1	F	27	27	86,2	0	22,9	3,4	X	X	X	X	X
2	M	42	26	102,9	0	20,4	3,5	X	X		X	X
3	M	45	23	98,8	0	31,0	3,9	X				
4	M	27	26	88,4	100	21,6	4,1	X	X	X	X	
5	F	32	20	103,9	0	20,1	4,6	X	X		X	
6	M	42	22	77,4	200	24,5	4,9	X	X	X		
7	M	36	22	118,1	0	27,2	6,0	X	X		X	
8	M	27	21	96,5	0	22,0	6,6	X				
9	F	27	22	108,5	400	20,1	6,7	X	X	X	X	
10	F	22	23	119,8	0	21,7	7,2	X	X		X	
11	M	27	27	98,2	0	25,4	7,8	X				
12	M	25	23	86,4	0	22,6	8,3	X				
13	F	44	26	94,3	400	27,2	8,6	X				
14	F	23	25	87,3	0	21,3	8,8	X				
15	M	48	22	91,9	400	21,1	9,4	X	X		X	X
16	F	25	21	82,1	0	25,6	9,5	X				
17	F	26	33	103,4	0	21,2	10,6	X				
18	F	40	24	90,1	400	22,5	11,4	X	X	X	X	
19	M	31	24	92,3	400	23,5	12,0	X				X
20	F	24	27	95,7	200	20,9	12,2	X	X		X	
21	M	22	20	92,7	400	20,3	14,1	X	X	X	X	
22	F	24	21	94,8	400	24,2	14,4	X	X	X		
23	M	30	24	84	400	28,9	15,2	X	X	X		
24	F	24	23	107,2	400	23,0	15,8	X	X		X	X
25	M	21	26	89,2	400	20,9	20,2	X				
26	M	49	25	114,5	0	24,2	20,3	X	X		X	
27	F	25	24	94,8	0	25,4	21,7	X			X	
28	M	39	29	95,1	200	27,2	23,7	X	X	X	X	
29	F	29	27	90,6	0	30,3	24,4	X	X		X	X
30	F	20	23	111,7	200	22,2	27,6	X			X	
31	F	22	20	99,1	0	20,5	33,2	X	X	X		
32	M	21	26	114,3	0	21,2	36,3	X	X		X	

BMI = body mass index, FEV_1 = forced expiratory volume in 1 second, ICS = inhaled glucocorticosteroid, MS = Mass Spectrometry, FACS = Fluorescence-activated cell sorting, HA = Hyaluronan, SDF-1 = Stromal -derived factor 1

Supplementary table 2. FACS antibodies used in the study.

	Conjugate	Product number	Dilution
CD34	PE	BD 550619	1:5
CD45	PE Cy7	BD 560915	1:40
CD11b	BV421	BD 562632	1:20
CXCR4 (CD184)	BV786	BD 741001	1:40
Isotype ctrl	BV786	BD 563732	1:40
CD3	PerCPCy5,5	BD 560835	1:20
CD14	PerCPCy5,5	BD 557923	1:10
CD19	PerCPCy5,5	BD 561295	1:20
CD20	PerCPCy5,5	BD 332781	1:10
CD294	PerCPCy5,5	BD 561660	1:20
CD16	BV510	BD 563830	1:20
CD115	AF647	BD 564945	1:40
COL-1	FITC	SB 1441-02	1:10
Isotype ctrl	FITC	SB 0104-02	1:10

BD: BD Bioscience, SB: Southern Biotech

Supplementary figure 1. Log2 protein intensity values, adjusted for the effect of sex, at baseline and 23 hours post allergen challenge. Individual patients are represented by unique colours and within patient change are highlighted by connecting lines between timepoints.

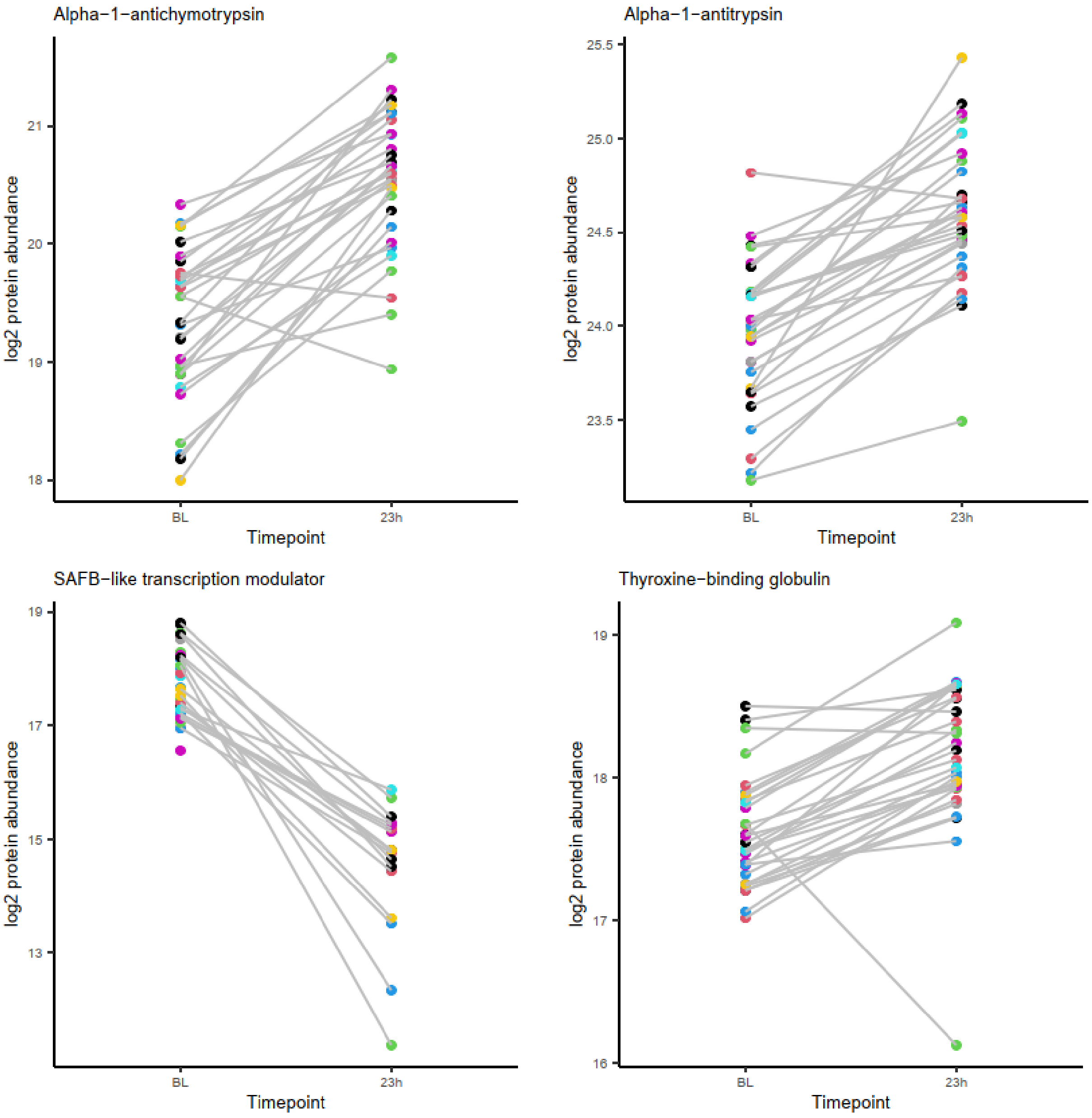

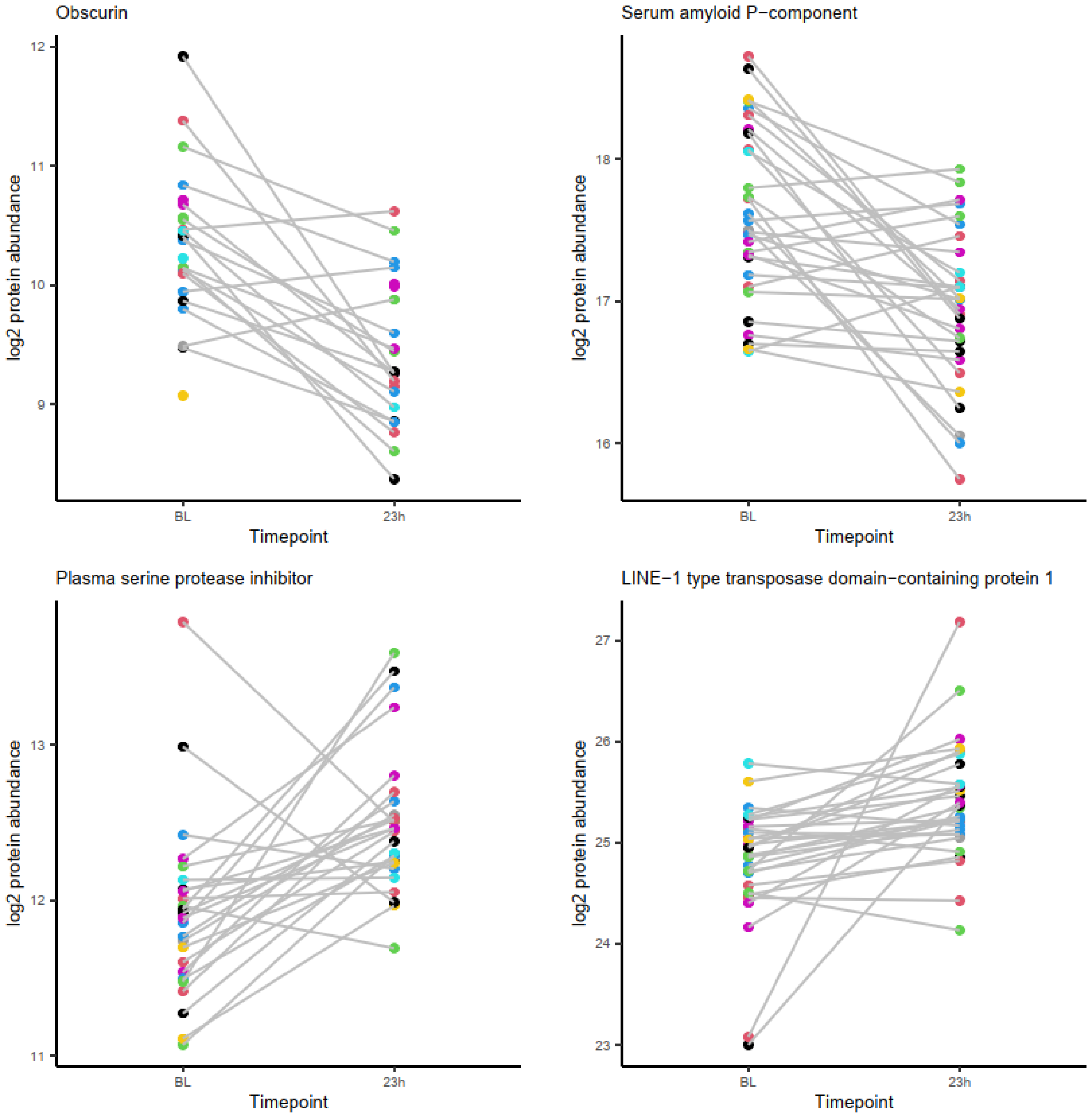
Obscurin
log2 protein abundance
12
11
10
9
BL
23h
Timepoint
Serum amyloid P−component
log2 protein abundance
18
17
16
BL
23h
Timepoint
Plasma serine protease inhibitor
log2 protein abundance
13
12
11
BL
23h
Timepoint
LINE−1 type transposase domain−containing protein 1
log2 protein abundance
27
26
25
24
23
BL
23h
Timepoint

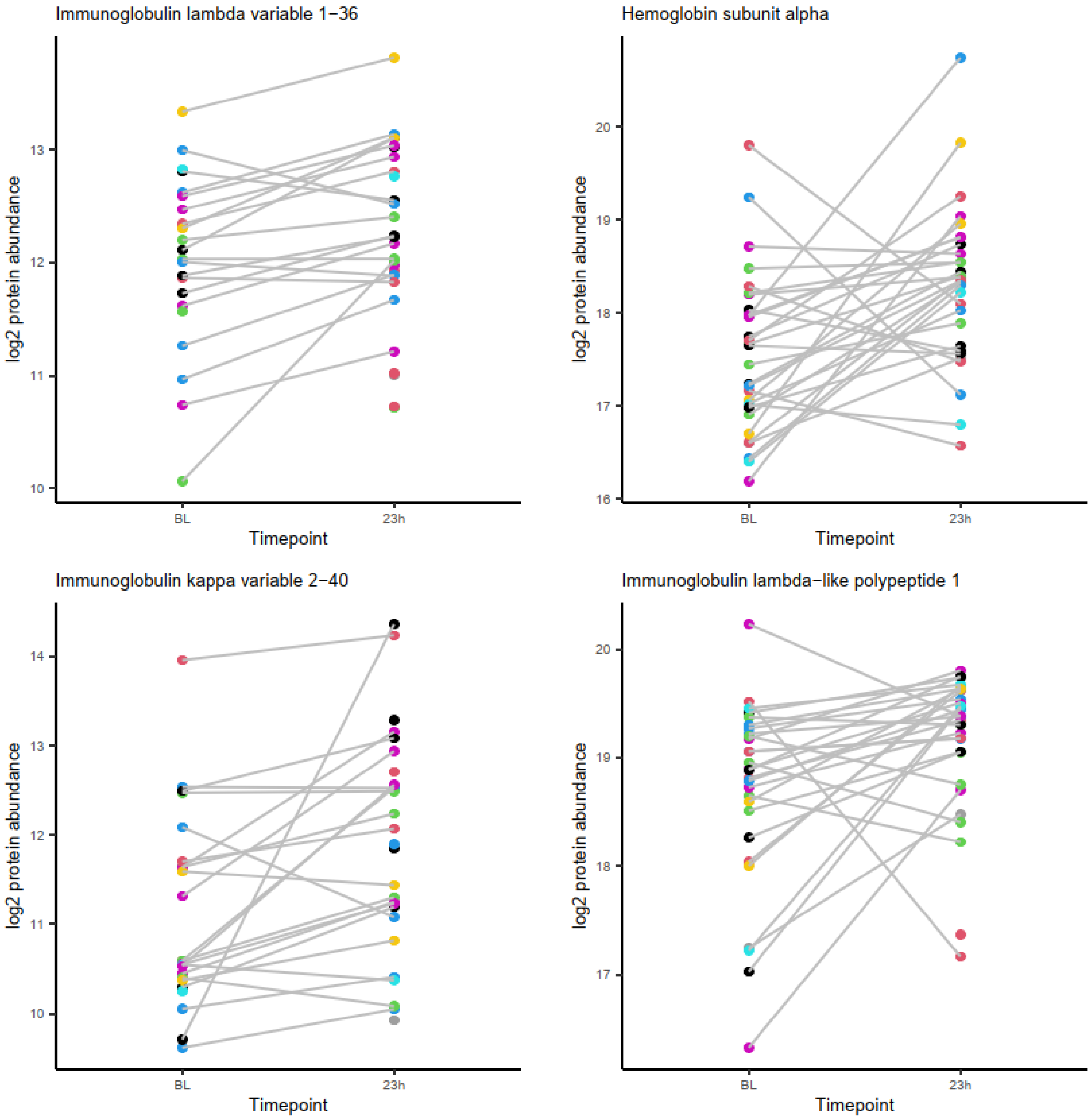
Immunoglobulin lambda variable 1−36
Hemoglobin subunit alpha
Immunoglobulin kappa variable 2−40
Immunoglobulin lambda−like polypeptide 1
log2 protein abundance
Timepoint
BL
23h

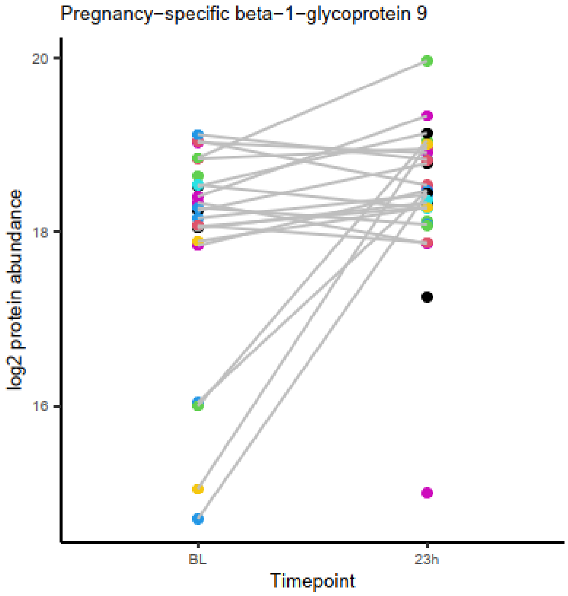
Pregnancy−specific beta−1−glycoprotein 9
log2 protein abundance
20
18
16
BL
23h
Timepoint

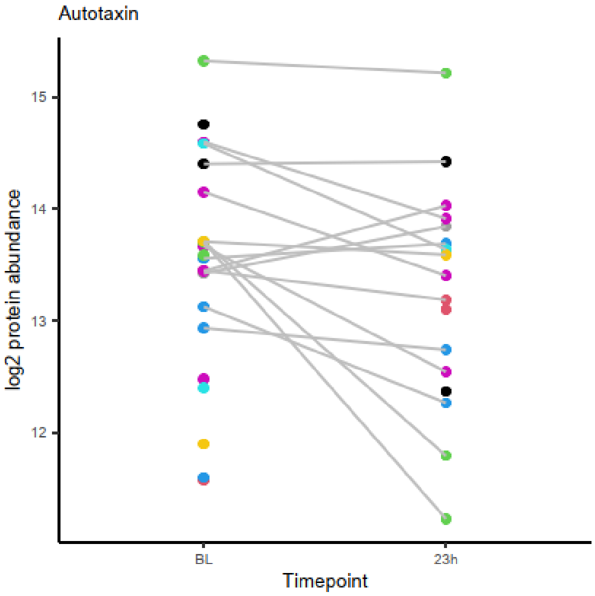
Autotaxin
log2 protein abundance
15
14
13
12
BL
23h
Timepoint

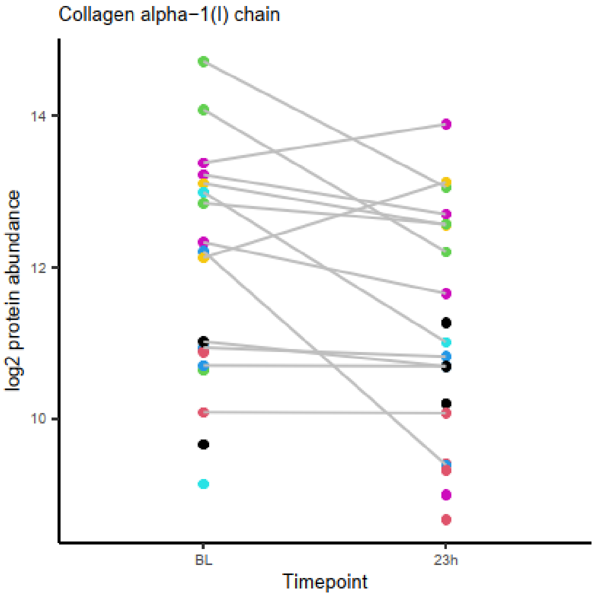
Collagen alpha−1(I) chain
log2 protein abundance
14
12
10
BL
23h
Timepoint

Supplementary figure 2. Change in protein abundance from baseline to 23 hours, plotted against the maximal drop in FEV_1 in % 4-8 hours post allergen challenge, representing the magnitude of the late phase response. All plotted proteins had a statistically significant (p<0,05) correlation to the drop in FEV_1 at 4-8 hours in the DEqMS analysis. The plotted values have been adjusted for the effect of sex, each data point represent one subject.

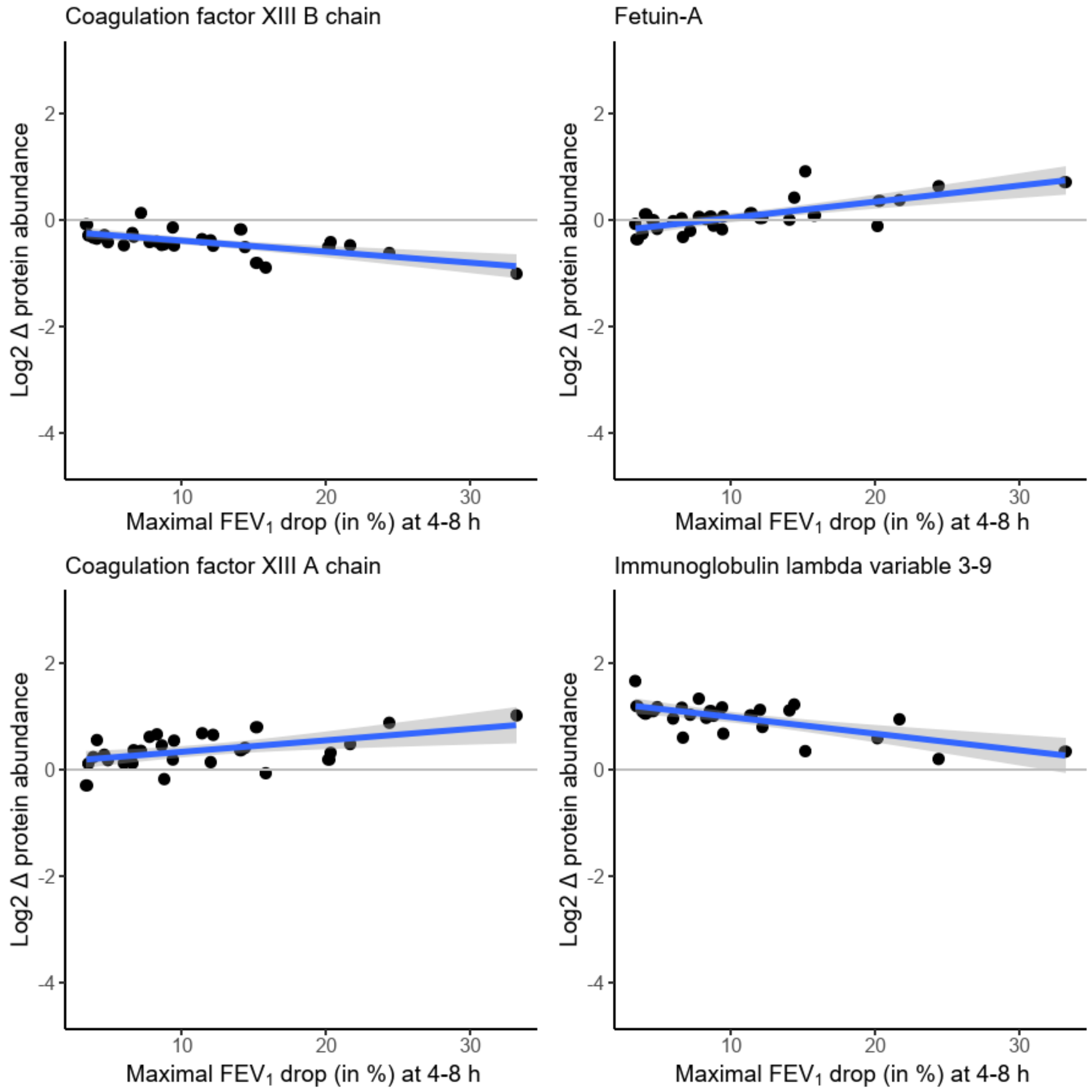

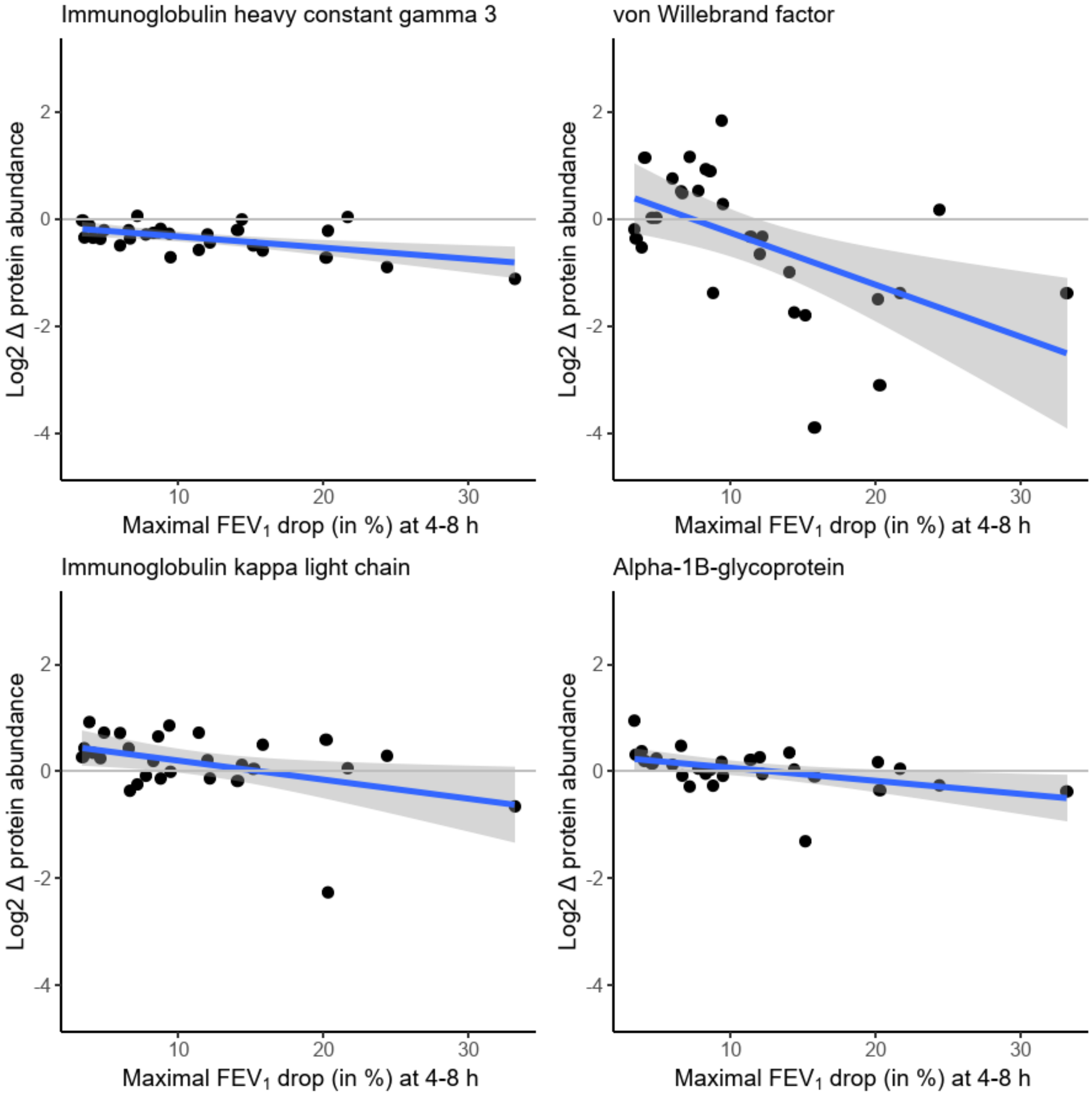
Immunoglobulin heavy constant gamma 3
von Willebrand factor
Immunoglobulin kappa light chain
Alpha-1B-glycoprotein
Log2 Δ protein abundance
Maximal FEV1 drop (in %) at 4-8 h
2
0
-2
-4
10
20
30

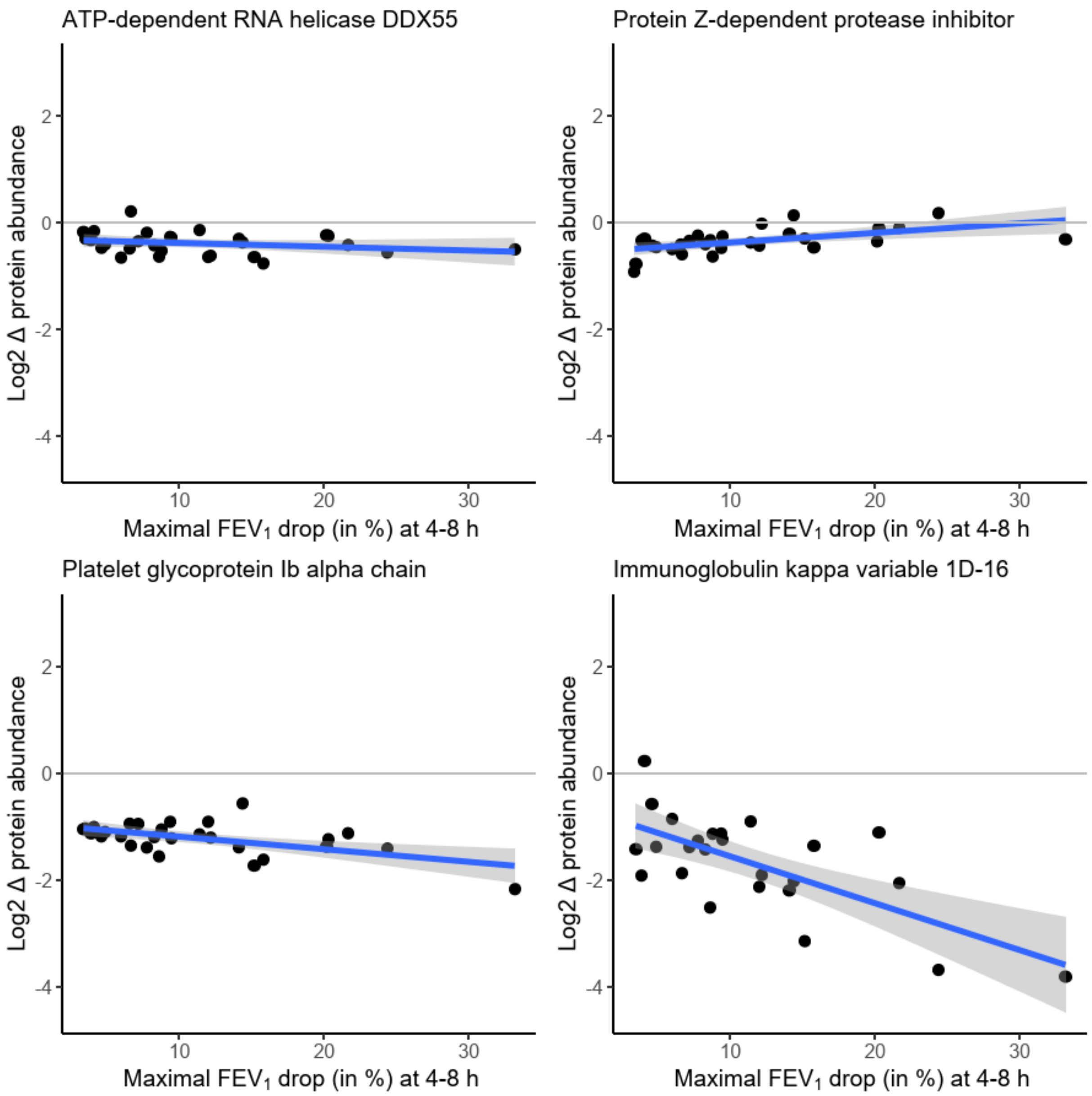

ATP-dependent RNA helicase DDX55
Protein Z-dependent protease inhibitor
Platelet glycoprotein Ib alpha chain
Immunoglobulin kappa variable 1D-16
Log2 Δ protein abundance
Maximal FEV1 drop (in %) at 4-8 h
2
0
-2
-4
10
20
30

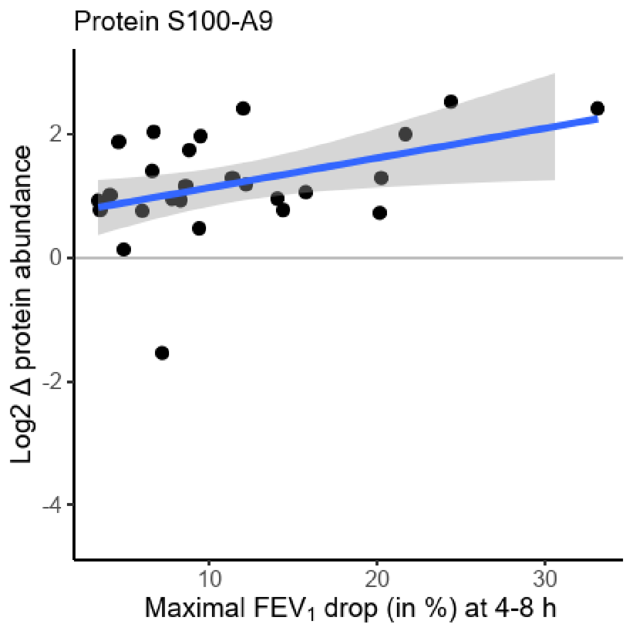
Protein S100-A9
Log2 Δ protein abundance
2
0
-2
-4
10
20
30
Maximal FEV1 drop (in %) at 4-8 h

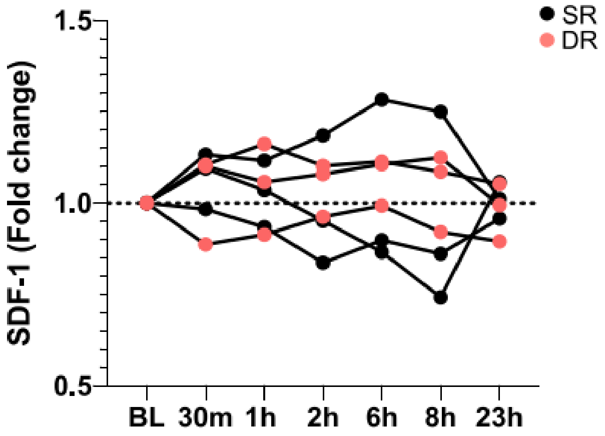

Supplementary figure 3. Temporal expression of SDF-1 in plasma.
